# Aerosolized Quercetin-Loaded Chia Seed Polysaccharide Nanoparticles: Design of Experiments and Machine-Learning-Guided Optimization for Enhanced Lung Cancer Cell Delivery

**DOI:** 10.3390/pharmaceutics18091095

**Published:** 2026-08-30

**Authors:** Sara Hasan, Seyedeh Negin Kassaee, Derek J. Richard, Nazrul Islam, Emad L. Izake

**Affiliations:** 1 School of Chemistry and Physics, Queensland University of Technology (QUT), Brisbane, QLD 4000, Australia; 2 Centre for Materials Science, Queensland University of Technology (QUT), 2 George Street, Brisbane, QLD 4000, Australia; 3 Cancer & Ageing Research Program, Centre for Genomics and Personalised Health, Queensland University of Technology (QUT), 60 Musk Ave, Brisbane, QLD 4059, Australia; derek.richard@qut.edu.au; 4 Pharmacy Discipline, School of Clinical Sciences, Faculty of Health, Queensland University of Technology (QUT), Brisbane, QLD 4000, Australia; seyedehnegin.kassaee@hdr.qut.edu.au (S.N.K.); nazrul.islam@qut.edu.au (N.I.)

**Keywords:** pulmonary nanomedicine, artificial neural network, cellular uptake, reactive oxygen species

## Abstract

**Background/Objectives:** The recent advances in pulmonary delivery have shifted the paradigm to the development of inhalable drug-loaded polysaccharide particles that can be positioned at the respiratory interface while reducing the systemic exposure. Quercetin has broad anticancer activity but remains difficult to translate because of its poor aqueous solubility, limited bioavailability and rapid metabolic loss. Here, we report quercetin-loaded chitosan/*Salvia hispanica* polysaccharide nanoparticles as a natural polyelectrolyte nanocarrier for pulmonary delivery in lung cancer. **Methods:** Central composite design (CCD) and artificial neural network (ANN) models were used for mapping the factors to responses. **Results:** The models displayed a higher predictive accuracy and optimization reliability for identifying the optimized formulation. The optimized nanoparticles showed a quasi-spherical morphology (mean hydrodynamic diameter = 331 ± 14.34 nm) with cationic ζ-potential of +36.3 ± 2.56 mV and polydispersity index of 0.15. The optimized formulation showed acceptable powder-flow characteristics, an encapsulation efficiency of 73.8 ± 0.73%, drug loading of 14.20 ± 0.22%, and biphasic release with sustained quercetin release over 48 h. In A549 and H460 cell lines, nanoencapsulation increased the antiproliferative effect of quercetin relative to the free compound, yielding lower IC_50_ values after 48 h of exposure. The nanoparticles showed greater suppression of wound closure, increased reactive oxygen species fluorescence and clear cellular uptake. Blank nanoparticles produced only limited effects. **Conclusions:** These results indicate that CCD/ANN-guided chitosan/*Salvia hispanica* polysaccharide nanoparticles provide a promising nanodelivery tool for quercetin delivery and enhanced in vitro activity in lung cancer cells, while the observed aerosol performance advocates further investigation of their pulmonary delivery potential.

## 1. Introduction

Lung cancer remains a leading cause of cancer-linked mortality globally. This chronic ailment accounts for more than 1.8 million deaths annually and shows a persistently lower survival rate despite advances in targeted therapies [1,2]. Chemotherapy, a central therapeutic route, continues to save lives but often gets hampered by its poor tumor specificity, rapid metabolic clearance and dose-limited side effects [3,4]. These barriers underline the critical unmet need for non-toxic delivery tools with potential to allow an increased intrapulmonary drug concentration while minimizing systemic burden [5]. These challenges have catalyzed the interest in discovering new nanoparticle-based delivery tools that can stabilize hydrophobic agents, thus improving their dispersibility and protection against metabolic pathways [6]. In this context, pulmonary delivery by inhalable nanoparticles has emerged as an attractive therapeutic paradigm in lung cancer [4]. These delivery carriers can evade the hepatic first-pass metabolism, promote the deposition within respirable lung regions, augment retention within tumor-bearing airways and achieve intracellular delivery in alveolar epithelium [7,8]. However, the formulation of biocompatible, biodegradable and economical inhalable nanoparticles remains a translational challenge [9].

Chia seed gum, *Salvia hispanica* L. seed-derived polysaccharide, has gained considerable attention in recent times owing to its unique physicochemical functionality. It is a high-molecular-weight anionic heteropolysaccharide containing xylose, glucose mannose and glucuronic acid. The presence of abundant hydroxyl and acidic sugar residues provides a strong water affinity, negative charge density and the ability to form hydrated polymer networks. These structural attributes underpin chia seed gum’s potential as a stabilizer, thickener, emulsifier, and encapsulating matrix. However, its use as polyelectrolyte complex with cationic polymer has received limited attention [10,11,12,13,14]. Amongst various cationic polysaccharides, chitosan has become relevant for drug delivery because of its cationic, biodegradable and structurally adaptable nature [15]. The presence of cationic amine functionalities enables it to interact with anionic polysaccharide to form nanoscale assemblies through electrostatic interactions. These complementary charge-driven interactions allow the formation of polyelectrolyte nanostructures with anionic polysaccharides such as chia seed polysaccharide under mild aqueous conditions [16,17]. Moreover, the development of nanoparticles is governed by complex interactions between formulation composition and processing conditions, making systematic optimization important for defining robust formulation–response relationships. Central composite design is commonly employed to quantify both individual and interactive effects of formulation variables whereas the artificial neural network provides a complementary computational approach capable of modeling complex and non-linear relationships between formulation inputs and critical quality attributes. The value of such data-driven approaches has also been demonstrated in pulmonary formulation development. The integration of central composite design with artificial neural networks may therefore provide a complementary experimental and predictive framework for optimizing nanoparticle formulations.

Quercetin has also been investigated in several inhalable delivery systems. Recent studies have evaluated pulmonary quercetin nanoparticles for lung cancer with respect to both aerosol performance and cellular toxicity and internalization, while inhalable quercetin-loaded alginate nanogels and modified silk fibroin nanoparticles have demonstrated alternative carrier strategies for pulmonary administration [18,19,20]. In this context, the present study provides a distinct inhalable quercetin nanoparticle-based platform based on chitosan and chia seed polysaccharide. The effects of excipient concentration and key processing parameters on particle size, encapsulation efficiency and drug loading were systematically investigated using central composite design while the resulting formulation–response relationships were further modeled using artificial neural networks. Following the optimization, the selected formulation was evaluated for aerosol performance, intracellular drug delivery, and anticancer activity in lung cancer cell lines.

## 2. Methodology

### 2.1. Chemicals

Quercetin (QTN, ≥95%) and low-molecular-weight chitosan (CtS, 150–250 kDa) were obtained from Merck Life Science Pty Ltd. (Bayswater, VIC, Australia). *Salvia hispanica* L. seeds were purchased from a local market. Ethanol (C_2_H_5_OH, ≥99%/analytical grade), sodium hydroxide (NaOH, ≥98%/analytical grade) and glacial acetic acid (CH_3_COOH, ≥99.7%/analytical grade) were all sourced from Merck Life Science Pty Ltd. (Bayswater, VIC, Australia). Hydrochloric acid (HCl, ≥37%), sodium chloride (NaCl, ≥99%), DCFH-DA (2′,7′-dichlorodihydrofluorescein diacetate) and phosphate-buffered saline (PBS, pH 7.4) were of analytical grade (Merck Life Science Pty Ltd., Bayswater, VIC, Australia) and used without further purification. Dialysis membranes (flat, 25 mm, MWCO = 12 kDa) were purchased from Merck Life Science Pty Ltd. (Bayswater, VIC, Australia). Deionized water (18.2 MΩ·cm at 25 °C) was used in all aqueous preparations.

### 2.2. Fabrication of Inhalable Nanoparticles

The *Salvia hispanica* L. polysaccharide (ShP) was extracted from seeds by hot water extraction [21]. Briefly, 100 g of seeds were washed, air-dried and subsequently hydrated in deionized water (500 mL) for 2 h to facilitate the seed coat swelling and initiate mucilage release. The hydrated seeds were then stirred at 1000 rpm for 30 min at 50 °C to promote mucilage dispersion while minimizing thermal degradation. The resulting mucilage was filtered via muslin cloth followed by mucilage precipitation by the addition of absolute ethanol at a 3:1 (*v*/*v*) ratio and was further characterized. The nanoparticles (NPs) were fabricated by using a modified ionic gelation approach by Kim et al. [21,22]. For this, QTN (0.01% *w*/*v*) solubilized in ethanol was added to CtS (0.02–0.05% *w*/*v*) in 1% acetic acid and stirred for 30 min at ambient temperature to obtain a uniform solution. The CtS/QTN solution was added dropwise to ShP solution (0.02–0.05% *w*/*v*) under continuous stirring. The resulting dispersion was homogenized (IKA ULTRA-TURRAX^®^ T25, IKA-Werke GmbH & Co. KG, Staufen im Breisgau, Germany) for 5 min and then subjected to sonication for 5 min (60% amplitude/20 W). The homogenized solution was freeze-dried by using a lyophilizer (Alpha 1–4 LD plus, Christ, Osterode am Harz, Germany), to obtain a dry NP powder. The powder was stored in amber vials until further characterizations.

### 2.3. CCD-Based Optimization

For the evaluation of the relationship between the formulation factors and responses, a central composite design based within a response surface methodology framework was used. The factor ranges were established by using already published data [21,23,24,25]. Four formulation variables (CtS (X_1_, 0.02–0.05% *w*/*v*), ShP (X_2_, 0.02–0.05% *w*/*v*), pH (X_3_, 4–6) and stirring speed (X_4_, 600–1400 rpm)) were investigated and optimized for their effects on three responses, particle size (Y_1_), encapsulation efficiency (Y_2_) and drug loading (Y_3_). A total of 56 experimental formulations were developed using Design-Expert^®^ software version 13 (DdE) (Stat-Ease Inc., Minneapolis, MN, USA). With the CCD, linear, interaction, and quadratic effects could be assessed in a systematic manner. The factor levels were all coded on a common dimensionless scale for model fitting, with the low, central, and high levels given values of −1, 0, and +1, respectively with axial points extended to ±2 (Table S1). Additionally, the experimental results were analyzed by fitting response surface models and then numerically optimizing these models. Three-dimensional response surface and interaction plots were created for the individual and combined effect of formulation variables on particle size and encapsulation efficiency using DdE software. A second-order polynomial equation was used to describe the relationship between the independent variables and each response:

$$Y\;=\;\beta_{0}\;+\;\sum_{i=1}^{4}\beta_{i}X_{i}+\;\sum_{i=1}^{4}\beta_{ii}X_{i}^{2}+\;\sum_{i<j}^{4}\beta_{ij}X_{i}X_{j}$$

where Y = predicted response, X_i_ and X_j_ = factors, β_0_ = intercept, β_i_ = linear coefficient, β_ii_ = quadratic coefficients and β_ij_ = two-factor interaction coefficient.

Moreover, analysis of variance (ANOVA) was used to assess the statistical significance of the model by using *p*-value, R^2^ (coefficient of determination) and CV% (coefficient of variation).

### 2.4. ANN-Based Optimization of Nanoparticles

The artificial neural network (ANN) was used to analyze the non-linear relationship between the formulation variables and the responses, a well-established approach in pharmaceutical formulation optimization [26,27,28]. The ANN analysis was performed using the same 56 formulations generated by the CCD-RSM design. The four independent variables were CtS concentration, ShP concentration, pH and stirring speed. Moreover, the CCD/RSM axial runs were retained during the ANN model development. Additionally, the responses, i.e., particle size (nm), encapsulation efficiency (%) and drug loading (%), were modeled independently. Each response was modeled by a separate multilayer perceptron regression built in Python 3.13 (scikit-learn version 1.8.0 ‘MLP Regressor’). The descriptive statistics are summarized in Table S4. Each ANN model contained four input neurons, a single hidden layer with hyperbolic tangent activation, and one regression output. The training set consisted of 45 components and a test set of 11 components. Moreover, both the input variables and response values were standardized using StandardScaler. The number of hidden neurons (between 2 and 10) was independently tested against each response (by repeated 5-fold cross validation) and the architecture with the highest mean cross-validated R^2^ within the training dataset was retained. The training–convergence curves were produced by an Adam-optimized network of the same architecture.

The approach of comparing the ANN with the ordinary multiple linear regression model, as done by Arulsudar et al., was adopted [29]. Additionally, both models were compared on the values of R^2^, RMSE and MAE for the test datasets. Variable importance was calculated by using two complementary approaches: permutation importance on the held-out test set and Garson’s connection weight algorithm. The predictions from all three trained networks were combined together via a desirability function with minimum particle size and maximum encapsulation efficiency and drug loading. The formulation with the highest desirability value was then selected by using differential evolution optimization within the practical formulation range. Moreover, a complementary Pareto analysis was subsequently performed using 2^21^ candidates with reproducible scramble Sobol candidate formulations to examine trade-offs among all three responses and for the identification of a Pareto-optimal formulation.

### 2.5. Physicochemical and Structural Characterization

The colloidal characteristics of QTN-loaded CtS/ShP NPs were evaluated to study their physicochemical properties. The hydrodynamic diameter and PDI were examined by dynamic light scattering whereas the zeta potential was determined by a Zetasizer Nano ZS, Malvern Instruments, Worcestershire, UK, via electrophoretic light scattering. The lyophilized samples (3 mg) were suspended in deionized water and sonicated for 5 min to ensure complete dispersion. All the measurements were taken in triplicate at ambient temperature. Moreover, the surface morphology was assessed by scanning electron microscopy (SEM; Zeiss Sigma VP Field Emission Scanning Electron Microscope, Carl Zeiss Microscope GmbH, Jena, Germany). The samples were placed on carbon-tape-mounted stubs and sputter-coated with a 5 nm layer of gold before imaging. Moreover, FTIR (Thermo iS5, Nicolet, Madison, WI, USA) was used to characterize the chemical features of the NPs and confirm the drug encapsulation within NPs.

### 2.6. Powder Density and Flow Properties

The flow properties of lyophilized CtS/ShP NPs were analyzed by bulk and tapped density, Carr’s index (CI), angle of repose and Hausner ratio (HR).

### 2.7. Drug Loading and Encapsulation Efficiency

The drug loading and encapsulation efficiency were determined by HPLC (Agilent HPLC Series 1100, Agilent Technologies, Santa Clara, CA, USA). For this, the NPs were centrifuged at 14,000× *g* for 20 min and the un-encapsulated quercetin (free drug) was collected. An aliquot of the supernatant was diluted with methanol (1:20 *v*/*v*), filtered through a 0.22 μm syringe filter and injected into the chromatograph. A reversed-phase C18 column (250 × 4.6 mm, 5 μm) was used with an isocratic mobile phase of acetonitrile: methanol: water (53: 27.5: 19.5, *v*/*v*/*v*) at a flow rate of 1 mL/min. The QTN was detected at 370 nm by UV/Vis. The concentration of QTN in the supernatant was quantified (n = 3), using the developed calibration plot and regression equation. The drug loading and encapsulation efficiency were determined by the following equations:

Encapsulation efficiency = (total drug − free drug/total drug) × 100

Drug loading (%) = (total drug − free drug/weight of the nanoparticles) × 100

### 2.8. In Vitro Aerosolization Performance

The aerosolization performance of NP powder formulations was evaluated using a twin-stage impinger (TSI) in accordance with the British Pharmacopoeia. A Breezhaler dry powder inhaler (Novartis Pharmaceuticals Pvt LtD., Macquarie Park, NSW, Australia) served as the delivery device. Stage 1 (S1) and Stage 2 (S2) of the TSI were filled with 7 mL and 30 mL of ethanol/water (50: 50 *v*/*v*), respectively. The airflow was maintained at 60 L/min with the Erweka pump and checked on the Fisher & Porter flow meter. About 20 mg of NP powder was inserted into size 3 hypromellose capsules (Vcaps^®^ Plus, Capsugel/Lonza, Basel, Switzerland) and dispersed into the TSI using Breezhaler. Each capsule was actuated for 5 s at 60 ± 5 L/min by vacuum pump (D-63150, Erweka, Langen, Germany) and verified with a calibrated digital flow meter (Fisher and Porter, Model 10A3567SAX, London, UK). The procedure was repeated 5 times (n = 5). After each actuation, the inhaler, S1 and S2 were rinsed separately with 50: 50 *v*/*v* ethanol and water and the filtrate was kept for analysis. The gravimetric assessment of the deposited NP mass was performed by filtering the collected washings through predried, preweighed 0.20 μm filter membrane and drying them at 60 °C until constant weight was reached. For determination of drug content, the washings were stirred at 37 °C for 48 h prior to HPLC quantification. Aerosolization efficiency was expressed in terms of recovered dose (RD = total NP amount collected from the inhaler and both TSI stages (S1 and S2)), emitted dose (ED) and fine particle fraction (FPF), calculated as:

ED = (S1 + S2)/RD × 100

FPF = S2/RD × 100

### 2.9. In Vitro Drug Release and Kinetics

The in vitro drug release from the QTN and QTN-loaded CtS/ShP NPs was evaluated using a dialysis membrane diffusion method performed in triplicate, adapted from a previously reported procedure with minor adjustments. Dialysis tubing (molecular cut-off 12 kDa) was preconditioned by soaking in deionized water for 24 h before use. An accurately weighed quantity of NP formulation, corresponding to 2 mg of QTN, was dispersed in an appropriate volume of medium and transferred into the prepared dialysis bag. The membrane was immersed in 50 mL of an ethanol/water mixture (1:1, *v*/*v*) used to maintain the sink condition medium in a beaker and maintained under continuous magnetic stirring at 100 rpm at 37 °C. At predetermined time points (0, 2, 4, 6, 8, 12, 24, 36, 48 h), 1 mL aliquots were withdrawn from the external release medium and immediately replaced with an equal amount of fresh medium to preserve constant volume and sink conditions throughout the study. The collected samples were quantified by high-performance liquid chromatography with detection at 370 nm. The kinetic release of pure drug and NPs was analyzed by using MATLAB R2024b. The cumulative release values were fitted into five models, namely, zero, first, Higuchi, Hixson–Crowell and Korsmeyer–Peppas models were used.

### 2.10. Cytotoxicity Studies

The H460 (ATCC HTB-177) and A549 (ATCC CCL-185) cell lines obtained from ATCC were maintained in RPMI 1640 supplemented with 10% fetal bovine serum at 37 °C in a humified 5% CO_2_ atmosphere. For subculture, monolayers were rinsed with PBS and detached using 0.5% trypsin for approximately 4 min. The enzymatic detachment was quenched with complete medium prior to reseeding. For viability assessment, cells were seeded at 1 × 10^4^ cells per well in white 96-well plates and allowed to adhere overnight. Cultures were then exposed to free QTN and QTN-loaded CtS/ShP NP formulations at QTN-equivalent concentrations of 400, 200, 100, 50, 25, 15 and 10 μg/mL for 24 and 48 h. The blank NPs were tested at corresponding NP concentrations. Cellular ATP content, as an index of metabolically active cells, was quantified using the CellTitre-Glo^®^ 2.0 Cell Viability Assay (Promega Corporation, Madison, WI, USA) according to the manufacturer’s instructions. Luminescence was measured with a PHERAstar FSX (BMG LABTECH, Ortenberg, Germany). Background signals were subtracted, and readings were normalized to untreated controls. The dose–response relationships were constructed and IC_50_ values were calculated.

### 2.11. Proliferation Assay

A549 and H460 cells (3 × 10^3^ cells/well) were seeded into 96-well plates and allowed to attach for 24 h. The culture medium was then replaced with fresh medium containing either QTN-loaded nanoparticles or free QTN at the 48 h NPs IC_50_-equivalent concentrations determined from the viability assay. Specifically, cells were treated with QTN-loaded nanoparticles and free QTN at 78.8 µg/mL (A549) and 56.5 µg/mL (H460). Untreated cells (medium only) served as controls. Cell proliferation was monitored as percentage confluency using the IncuCyte live-cell imaging system (Essen Biosciences, Dandenong South, VIC, Australia), with images acquired at 12 h intervals for up to 96 h. Confluence values were calculated using IncuCyte analysis software 2020C Rev1 and expressed relative to the untreated control.

### 2.12. Cell Migration

Cell migration was examined by a scratch wound assay to compare QTN with its NP formulation on H460 and A549 cell lines. The cells were seeded in 6-well plates at 2 × 10^5^ cells per well in 2 mL of complete medium and grown to 90–100% confluency. Marks were placed beneath each well to relocate the same field during imaging. After aspirating the medium, 0.5 mL PBS was added and a linear scratch was made across the monolayer with a sterile 200 uL pipette tip, perpendicular to the mark. Loose cells were removed by gentle PBS washing, leaving a clean wound edge. The PBS was then aspirated and 0.5 mL treatment solution was immediately added to prevent drying. The quercetin and drug-loaded nanoparticles were tested at IC_50_ concentrations, with untreated wells as controls. The images were taken every 24 h at the marked positions using optical microscopy to follow wound closure and cell migration.

### 2.13. ROS Generation

The intracellular ROS in H460 and A549 cell lines were measured with DCFH-DA. The cells were plated in 96-well plates at 5 × 10^3^ cells per well and grown for 24 h in complete medium to reach ~70% confluence. The medium was removed and cells were exposed for 6 h to QTN and QTN-loaded NPs at their respective IC_50_ values at 37 °C in 5% CO_2_. The treatment solution was replaced with 100 μL of 20 μM DCFH-DA and the plates were kept in the dark for 30 min to allow the probe to react inside the cells. The wells were washed three times with PBS to remove excess dye. Fluorescence was then read at 485 nm (excitation) and 520 nm (emission) as an indicator of intracellular ROS activity.

### 2.14. Cellular Uptake of NPs

The cellular uptakes studies were carried out on A549 (ATCC CCL-185) and H460 (ATCC HTB-177) cell lines, obtained from ATCC (Manassas, VA, USA). The H460 and A549 cell lines were seeded in 6-well glass-bottom plates (2 × 10^5^/well) in complete RPMI and left for 24 h. Free rhodamine and rhodamine-loaded NPs (100 µg/mL) were then added and incubated for 4 h. The cells were washed 3× with cold PBS, fixed with 4% PFA (20 min), permeabilized (0.2% Triton X-100, 30 min) and blocked with 1% BSA at 4 °C. The nuclei were stained with DAPI. The imaging was performed on a Nikon confocal microscope (20×) (Nikon Instruments Inc., Tokyo, Japan). The excitation and emission wavelengths were set at 540–560 nm and 570–590 nm respectively.

### 2.15. Statistical Analysis

Triplicate independent experiments were performed, except where stated otherwise. Data are presented as mean ± SD. A *p*-value < 0.05 was considered statistically significant. Statistical analysis was performed by analysis of variance (ANOVA) followed by Tukey’s post hoc test.

## 3. Results and Discussion

### 3.1. CCD-Based Modeling

A CCD design coupled with RSM was utilized to systematically study the effects of processing variables on the CtS/ShP NP attributes. In the present study, 56 runs were generated by the DdE software. Across the experimental design, particle size (nm) ranged from 348.2 to 404 nm, whereas encapsulation efficiency (%) and drug loading (%) varied from 64.31 to 76.64% and 7.77 to 15.46%, respectively. The responses observed showed that the physicochemical properties of the nanoparticles were sensitive to formulation composition and processing conditions.

Experimental data were well fitted by the second-order quadratic polynomial models and the statistical significance of each model was tested and also the adequacy of the model was checked by analysis of variance (ANOVA). The quadratic models were all statistically significant for the three responses (*p* < 0.0001). The coefficients of determination for particle size, encapsulation efficiency and drug loading were 0.9730, 0.9932 and 0.9908, respectively, indicating that the models explained the responses’ variability, ranging from 97.30% to 99.32%. The corresponding adjusted (R^2^) values were 0.9638, 0.9909, and 0.9876, while the predicted (R^2^) values were 0.9500, 0.9875, and 0.9822, respectively. The closeness of the adjusted values to the predicted values (R^2^) also showed good agreement between predictive ability and model fit. The CV% values were low for particle size (0.68%), encapsulation efficiency (0.42%), and drug loading (1.75%), suggesting low residual variation, compared to the size of the measured responses. The polynomial equations for all the responses were generated by the software.

Particle size = 353.475 − 8.48125X1 + 2.52X2 + 5.27X3 + 2.65X4 + 0.615X1 X2 − 0.85X1 X3 − 0.72X1 X4 + 0.52X2 X3 − 0.12X2 X4 + 1.47X3 X4 + 7.87X1^2^ + 5.10X2^2^ + 5.98X3^2^ + 3.85X4^2^

Encapsulation efficiency = 75.99 + 2.11X1 − 0.56X2 − 1.05X3 − 0.49X4 − 0.22X1 X2 + 0.205X1 X3 + 0.06X1 X4 − 0.047X2 X3 − 0.084X2 X4 − 0.049X3 X4 − 1.86X1^2^ − 1.49X2^2^ − 1.32X3^2^ − 1.017X4^2^

Drug loading = 15.13 + 1.304X1 − 0.33X2 − 0.63X3 − 0.34X4 − 0.16X1 X2 + 0.09X1 X3 + 0.12X1 X4 − 0.048X2 X3 + 0.03X2 X4 − 0.085X3 X4 − 1.14X1^2^ − 0.86X2^2^ − 0.74X3^2^ − 0.62X4^2^

**Figure 1:**
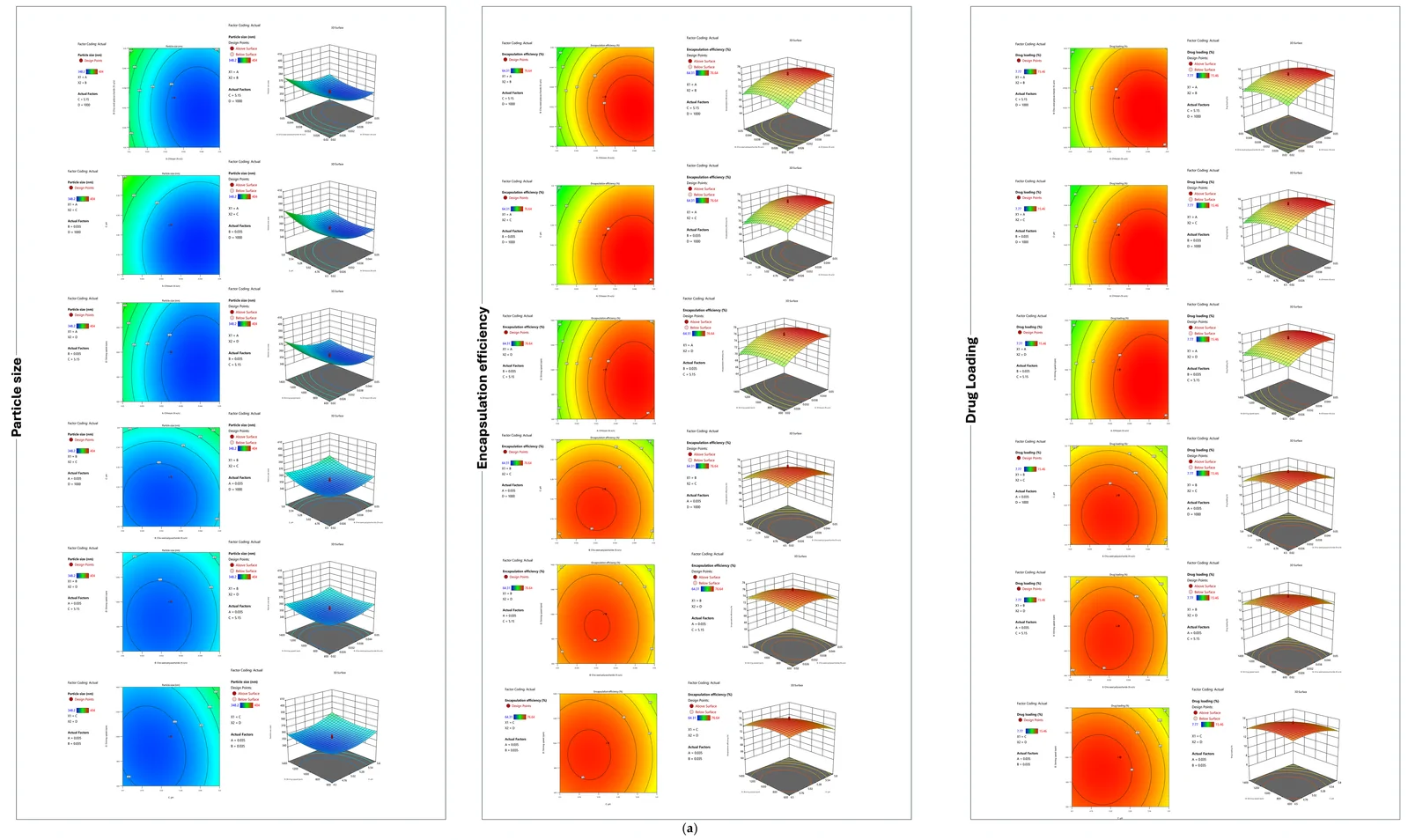

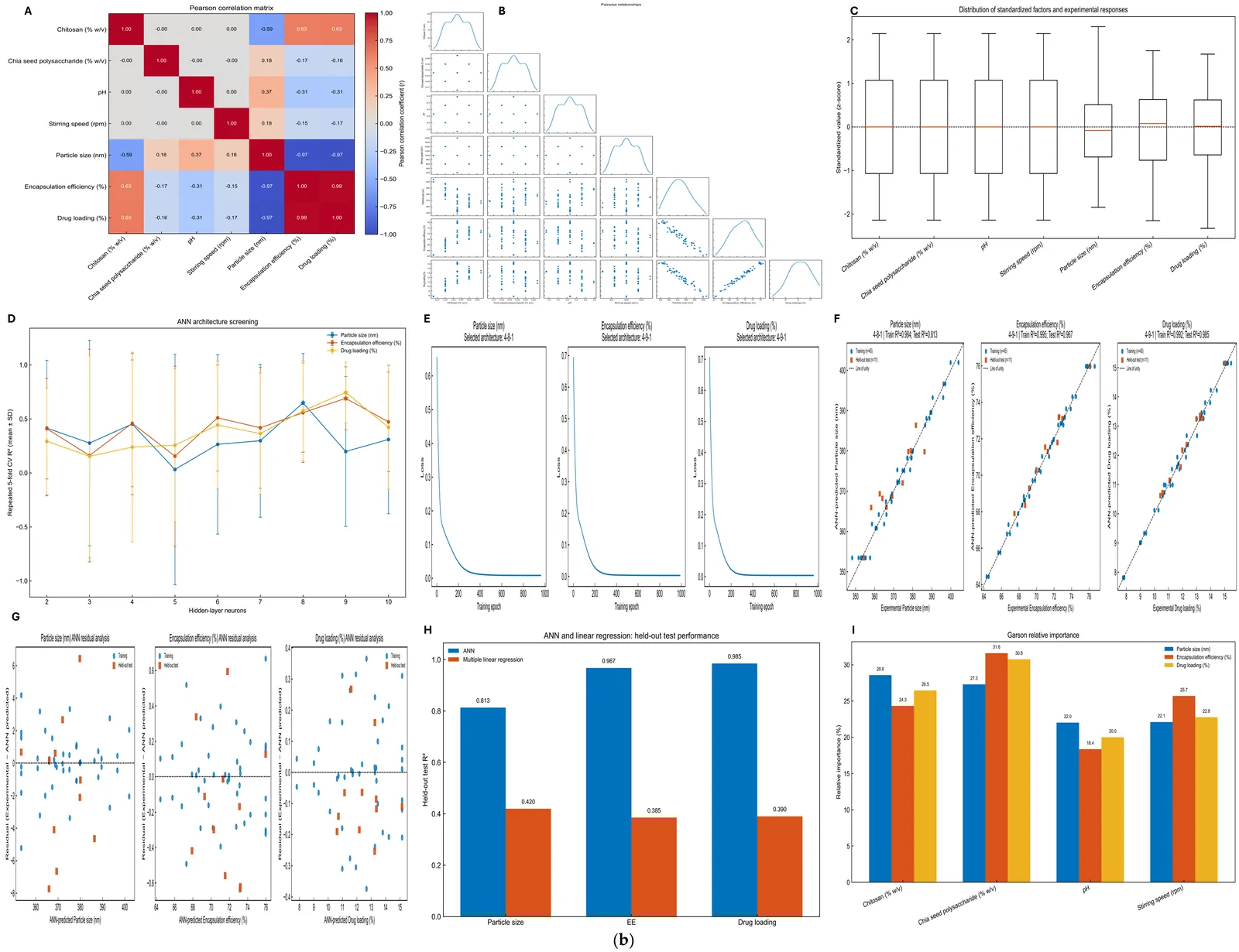
(**a**) 3D response-surface and two-dimensional contour plots. (**b**) ANN modeling outputs illustrating the relationship between factors and responses: (**A**) Pearson correlation matrix; (**B**) pairwise distribution and correlation plots; (**C**) distribution of standardized formulation factors and responses; (**D**) ANN architecture screening; (**E**) ANN training loss curves for responses; (**F**) experimental versus ANN predicted values for the responses; (**G**) residual plots; (**H**) comparison of ANN and linear-regression holdout test performance; and (**I**) comparison of model generalization performance.

The three-dimensional response-surface and two-dimensional contour plots (Figure 1a) were used to illustrate the effects of individual factors and their interactions. The concentration of CtS had the greatest effect on particle size with a negative linear effect suggesting a decrease in size with increasing concentrations of CtS, whereas the positive quadratic effect suggested a pronounced curvature in this relationship. Significant non-linear interactions between pH and stirring speed were also observed, which all also contributed to the variance in particle size observed.

Increasing the concentration of CtS resulted in improved encapsulation efficiency up to an intermediate optimum, however, increasing the concentration of ShP, pH and stirring rate of the system resulted in decreased encapsulation efficiency. Significant interactions between significant CtS/ShP and between CtS/pH suggested that drug entrapment was controlled by the interaction of polymer composition and the formulation pH. Similar patterns were seen regarding drug loading, with the greatest positive linear loading contribution being from CtS, with ShP, pH and stirring speed contributing comparatively negatively. The negative quadratic coefficients were observed for each of the factors, indicating a response curvature with maximum encapsulation efficiency and drug loading occurring within the intermediate levels of formulation and not at the extreme factor levels.

### 3.2. ANN Modeling

ANN models were used to capture the relationships between factors and responses. The particle size achieved a mean CV R^2^ = 0.6491 ± 0.4570 with a 4–8–1 architecture of the model, whereas the encapsulation efficiency and drug loading were best characterized by a 4–9–1 architecture of the model with mean CV R^2^ of 0.6908 ± 0.2918 and 0.7459 ± 0.2817, respectively. The screening of architecture was performed over 4–2–1 to 4–10–1 network range by using a repeated 5-fold cross-validation with five repeats. The coefficient of determination for particle size prediction was found to vary substantially across the candidate architectures, with mean CV R^2^ ranging from 0.0318 to 0.6491, compared with maximum mean CV R^2^ values of 0.6908 for encapsulation efficiency and 0.7459 for drug loading (Figure 1(bD)). Additionally, the relatively larger cross-validation variability for particle size was consistent with its lower generalization performance on the independent held-out test set.

#### 3.2.1. Training and Independent Test Performance

The predictive ability of the three ANNs is summarized in Figure 1(bF) (predicted vs. actual, training and held-out test sets) and Figure 1(bG) (corresponding residuals). The particle-size model displayed a training R^2^ of 0.9839 (RMSE = 1.77, MAE = 1.25) and a held-out test R^2^ of 0.8130 (RMSE = 4.24, MAE = 3.35). Residuals for particle size (Figure 1(bG)) were distributed around zero without an obvious trend across the predicted range, however, the larger test errors compared with the training set indicate lower generalization for this response. The drug-loading model also generalized well, with a training R^2^ of 0.9925 (RMSE = 0.175, MAE = 0.132) and a held-out test R^2^ of 0.9848 (RMSE = 0.163, MAE = 0.149) (Figure 1(bF)). The encapsulation-efficiency network also displayed a strong predictive performance (training R^2^ = 0.9949; RMSE = 0.236, MAE = 0.180) and a held-out test R^2^ of 0.9674, RMSE of 0.414 and MAE of 0.353. To assess whether the additional complexity of the ANN models was justified, each ANN model was compared with a multiple linear regression model using the same training and held-out test sets (Figure 1(bH)). The ANN models showed superior held-out predictive performance for all three responses on the test set: particle size (R^2^ = 0.813 vs. 0.420), encapsulation efficiency (R^2^ = 0.967 vs. 0.385) and drug loading (R^2^ = 0.985 vs. 0.390).

#### 3.2.2. Sensitivity Analysis

The relative influence of formulation factors on ANN predictions was evaluated by using the permutation importance technique and Garson’s algorithm (Table 1, Figure 1(bI)). Polymer concentrations were important predictors in both methods, although the polymers were ranked differently between the methods. The concentration of CtS was identified as the dominant variable by permutation importance by the prediction of particle size, contributing 53.3%, as well as predicting encapsulation efficiency (49.1%) and drug loading (49.7%). Moreover, Garson’s approach revealed a more balanced distribution of the four formulation variables. For particle size, the relative importance values were 28.6% for CtS concentration, 27.3% for ShP concentration, 22.0% for pH, and 22.1% for stirring speed. For encapsulation efficiency, the most influential variables were ShP concentration (31.6%), stirring speed (25.7%), CtS concentration (24.3%), and pH (18.4%). A similar pattern was observed with drug loading, for which the concentration of ShP (30.8%) was the most significant, followed by the concentration of CtS (26.5%), stirring speed (22.8%), and pH (20.0%). Overall, the permutation analysis identified that CtS concentration is the most important predictive factor for all three responses, whereas Garson’s analysis showed a more balanced contribution of all four formulation variables to ANN predictions.

#### 3.2.3. Optimized Formulation

A multiple-response optimization was performed independently using CCD/RSM models and the ANN models. For both approaches, the particle size was minimized while maximizing the encapsulation efficiency and drug loading. The optimized formulation from multi-response optimization was generated by DdE with 0.04% *w*/*v* CtS and 0.03% *w*/*v* ShP, at pH 4.9 and 900 rpm, with the predicted particle size, encapsulation efficiency, and drug loading values of 349.67 nm, 76.87%, and 15.6%, respectively, and an overall desirability of 0.99. ANN-based Pareto optimization identified a closely comparable optimized formulation comprising 0.04% *w*/*v* of CtS and 0.03% *w*/*v* ShP at pH 5.09 and 785 rpm was quite close (with responses of 352.6 nm, 76.5%, and 15.5%, respectively, and a desirability of 0.97). For experimental validation, the formulation was prepared using 0.04% CtS, 0.03% ShP, pH 4.9, and 900 rpm, yielding a particle size of 331 ± 14.3 nm, encapsulation efficiency of 73.8 ± 0.73%, and drug loading of 14.20 ± 0.22%. The comparison between predicted and experimentally validated optimized formulations is presented in Table 2. The conditions of the DdE experiments were selected for experimental preparation as they yielded the highest overall desirability. Both modeling approaches therefore provide complementary support for the identification of the optimal formulation region [30,31,32].

### 3.3. Physicochemical Characterization of Optimized Nanoparticles

**Figure 2:**
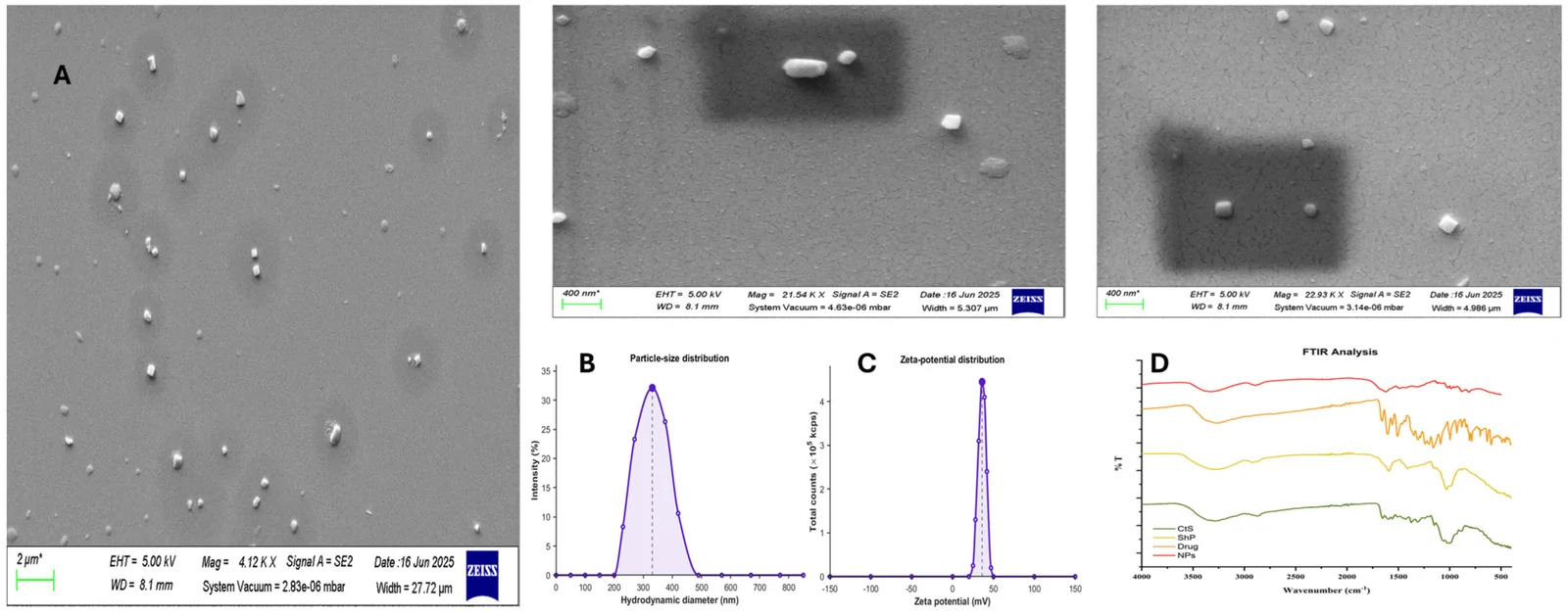
Physicochemical properties of QTN-loaded CtS/ShP NPs. (**A**) Representative SEM images showing predominantly spherical particles. (**B**) DLS hydrodynamic size distribution of NP dispersion. (**C**) Zeta potential distribution of QTN-loaded CtS/ShP NPs. (**D**) FTIR characterization of excipients, QTN, blank and QTN-loaded NPs. Where * is a software generated calibration index.

The particle size and surface properties of QTN-loaded CtS/ShP NPs were examined with a focus on inhalation suitability. The DLS measurements showed that the average hydrodynamic diameter (Z-average) of the QTN-loaded CtS/ShP NPs was ~331 ± 14.34 nm (PDI = 0.15). The ELS measurement indicated that the ζ-potential of the QTN-loaded CtS/ShP NPs was found to be +36.3 ± 2.56 mV (Figure 2C). The positive surface potential is consistent with a CtS-rich interface as excess protonated amines on CtS outweigh the anionic contribution of ShP gum. This provides a charge-mediated stabilization and a relatively narrow dispersed population (PDI < 0.15). Moreover, the SEM images of the QTN-loaded CtS/ShP NPs revealed that the NPs have discrete, quasi-spherical morphology with smooth, continuous surfaces. The size distribution of the nanoparticles was uniform, with an average nanoparticle diameter of 300 nm, which is in close agreement with DLS size measurements (Figure 2) [22,33,34].

Moreover, the NPs were characterized by FTIR (Figure 2D). The FTIR spectra of CtS, ShP, and QTN, blank NPs, and QTN/CtS/ShP NPs identified the characteristic functional groups of all components and intermolecular interactions inside the nanoparticles. The changes in the OH, amide I, and carboxylate bands of the blank CtS/ShP NPs signified the presence of hydrogen bonding and electrostatic interactions between ShP carboxylate groups and protonated CtS amines. Further displacement of these bands in QTN/CtS/ShP NPs, as well as low residual quercetin peaks, indicated drug encapsulation. The weak signal attenuation in the 1100–800 cm^−1^ region was consistent with changes in the hydrogen bond formation within the nanocarrier matrix. No new absorption bands were identified, affirming that the encapsulation was achieved via non-covalent interactions [6,21,35].

### 3.4. Powder Density and Flow Properties

Powder packing behavior is a practical determinant of capsule emptying, metering reproducibility and de-agglomeration in dry powder inhalers. Because nanostructured powders often exhibit increased cohesion due to high surface energy and van de Waals attraction, bulk and tapped densities were measured and used to derive Carr’s compressibility index (CI) and Hausner ratio (HR) alongside the angle of repose. The blank NPs gave CI 14.39% and HR 1.17 while QTN-loaded NPs gave CI 13.10% and HR 1.15, both within commonly cited acceptable thresholds for pharmaceutical powders (CI ≤ 20–25%; HR ≤ 1.25). Angles of repose were 30° (blank) and 28.6° (QTN-loaded NPs), supporting comparable flow behavior [36,37].

### 3.5. Drug Loading and Encapsulation Efficiency

The CtS/ShP NPs’ ability to incorporate QTN was assessed by the determination of encapsulation efficiency and drug loading. The optimized QTN-loaded CtS/ShP NPs displayed an encapsulation efficiency of 73.8 ± 0.73% and a drug loading of 14.20 ± 0.22%, indicating a substantial QTN encapsulation with NPs [7,38,39].

### 3.6. In Vitro Aerosolization Performance

**Figure 3:**
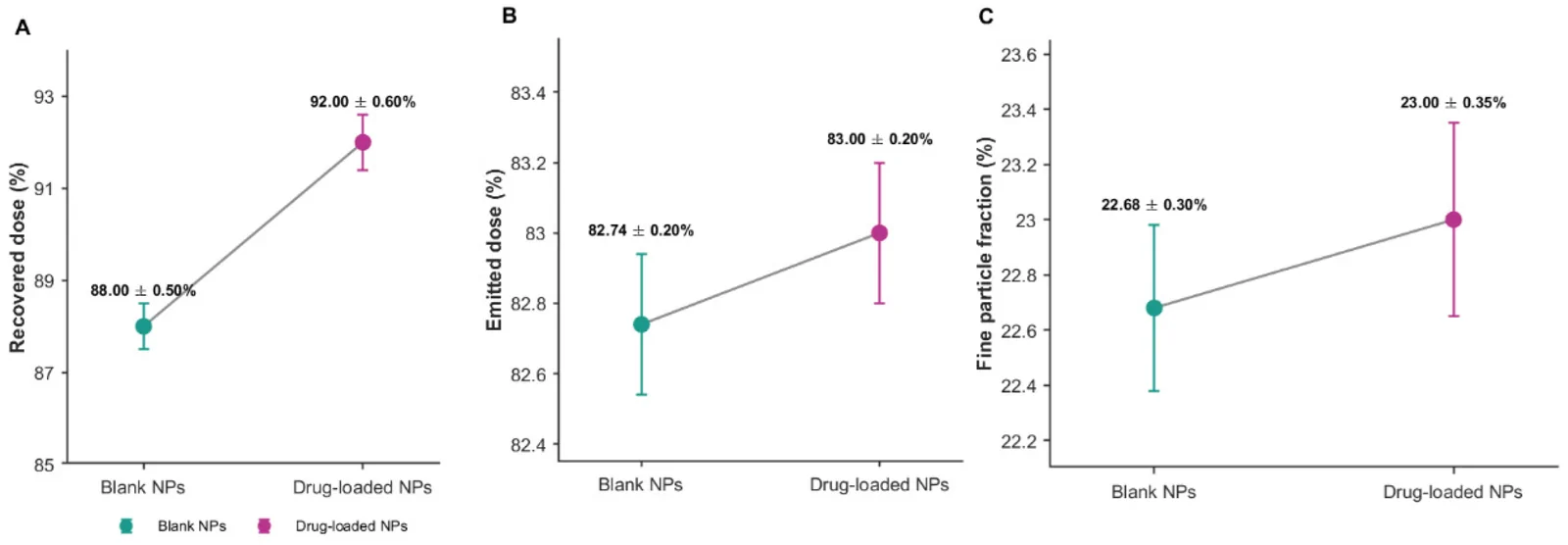
In vitro aerosolization performance of blank and drug-loaded chitosan/chia seed gum nanoparticles: (**A**) recovered dose (%); (**B**) emitted dose (%); and (**C**) fine particle fraction (%).

The aerosolization performance of blank and QTN-loaded CtS/ShP NPs was determined by using RD%, ED%, FPF and FPD by a twin-stage impinger (Figure 3). An efficient aerosol dispersion was shown by both the blank and drug-loaded NPs across both impinger stages with significant RD%, ED% and FPF% values. A slight improvement in aerosolization profile was observed in drug-loaded NPs in comparison to blank NPs, demonstrating that drug loading did not adversely affect the aerosol performance. Moreover, the QTN-loaded CtS/ShP NPs showed a fine particle dose of 417.7 µg, implying substantial delivery of active compound to respirable stages of the impinger. The observed slight improvement in aerosolization of the QTN-loaded CtS/ShP NPs over the blank nanoparticles could be attributed to the changes in the particle density and surface properties after the drug incorporation. Overall, these results show that the designed QTN-loaded CtS/ShP NPs have appropriate aerodynamic characteristics to deliver medications to the lungs [5,9].

### 3.7. In Vitro Drug Release and Kinetics

**Figure 4:**
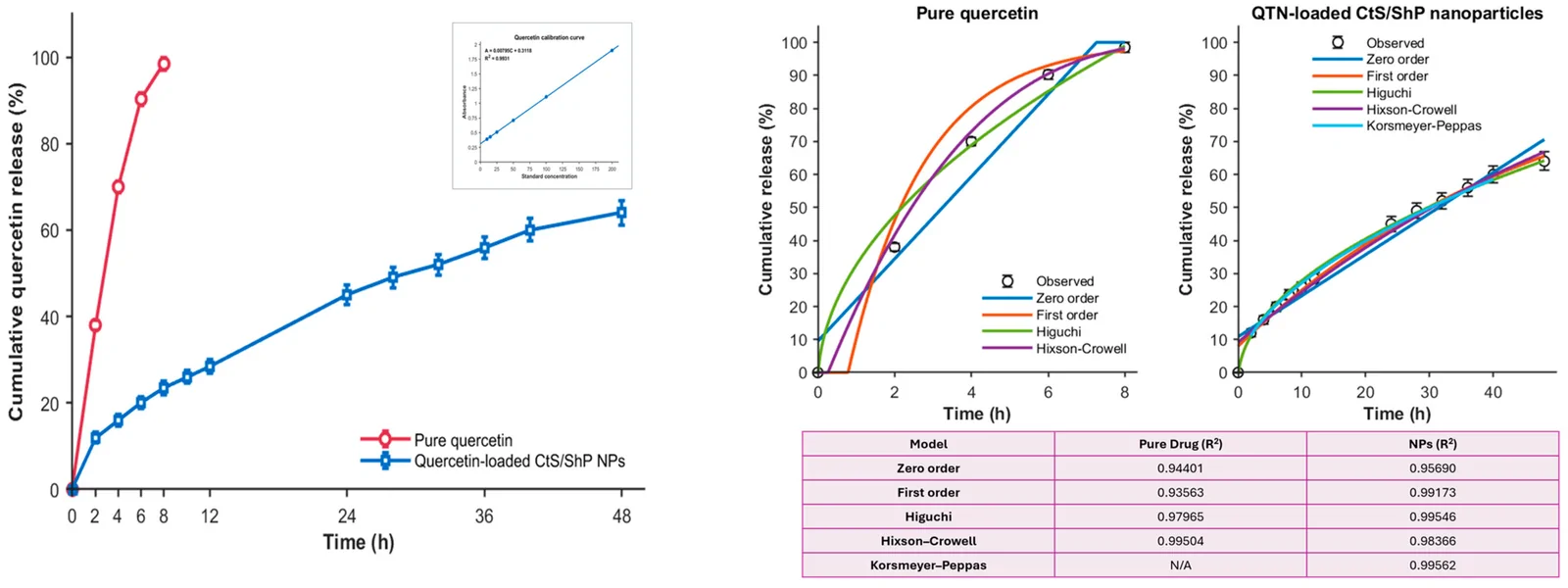
In vitro drug release profile of QTN-loaded CtS/ShP NPs and corresponding calibration curve over 48 h.

As depicted in Figure 4, QTN-loaded NPs showed a biphasic release pattern, encompassing a rapid initial release followed by a sustained release. A moderately early release was observed within the first two hours, which may be attributed to the QTN fraction located at the carrier’s surface. Consequently, the release rate declined gradually, reaching ~30% in 12 h, ~45% at 24 h and ~64% at 48 h. This indicated that QTN transport became increasingly controlled by diffusion/relaxation via the CtS/ShP matrix. This sustained release is consistent with ionic complexation between cationic CtS and anionic ShP, which forms a cross-linked network that increases tortuosity while restricting polymer-chain mobility. This mechanism has been widely reported for CtS-based polyelectrolyte-complex-based nanoparticulate systems. This release profile advocates the efficacious controlled release potential of CtS/ShP NPs for QTN at 37 °C under sink conditions. Additionally, the in vitro release values were fit into five kinetics models. The pure QTN displayed a rapid release (98.45% within 8 h) and was best described by the Hixson–Crowell model (R^2^ = 0.99504, k*_HC_* = 0.0947). Contrastingly, QTN-loaded CtS/ShP NPs were best described by the Higuchi model with R^2^ = 0.99546 and k*_H_* = 9.642, showing a substantial diffusion-controlled contribution to QTN release through the hydrated matrix. Additionally, the initial release was best fit to the Korsmeyer–Peppas model with R^2^ = 0.99562 and n = 0.55, i.e., anomalous transport, indicating that the QTN release from NPs was governed by a combined effect of diffusion via hydrated matrix and polymer-chain relaxation [6,35,40,41].

### 3.8. Cytotoxicity Studies

**Figure 5:**
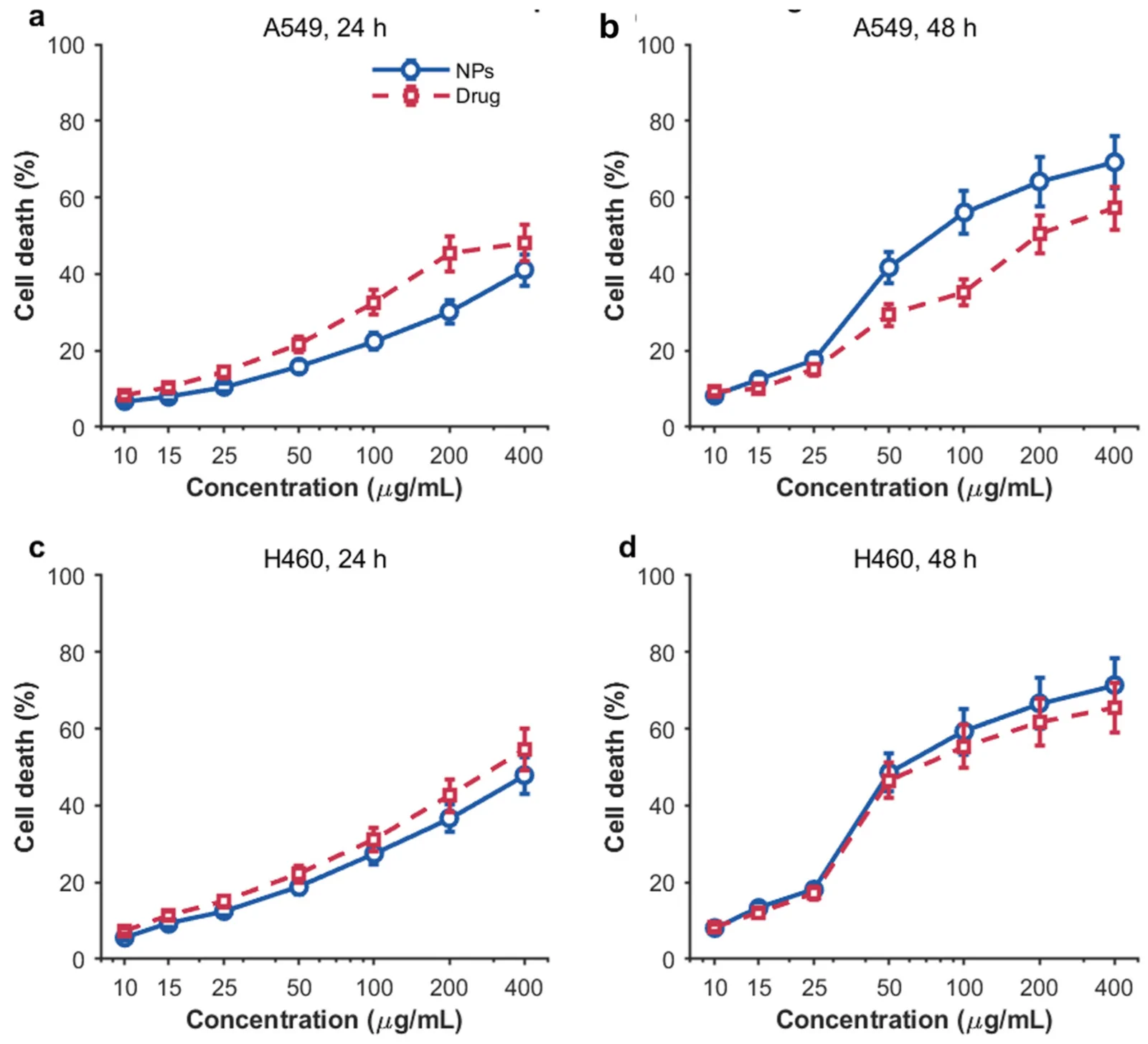
Cytotoxic activity of QTN-loaded NPs compared with free QTN in lung cancer cells (A549 and H460), 10–400 µg/mL for 24 and 48 h: (**a**) A549 cells at 24 h; (**b**) A549 cells at 48 h; (**c**) H460 cells at 24 h; and (**d**) H460 cells at 48 h.

The cytotoxic activity of blank and QTN-loaded NPs was evaluated in A549 and H460 lung cancer cells after 24 and 48 h exposure and compared with free QTN at the same nominal concentrations (10–400 µg/mL) (Figure 5). The free QTN and QTN-loaded CtS/ShP NPs were compared at drug-equivalent concentrations whereas the blank NPs were tested at corresponding NP concentrations. The blank NPs showed negligible cytotoxicity at all concentrations. In both models, viability decreased with increasing concentration and longer incubation, with the most pronounced cytotoxicity observed at 48 h. In A549 cells at 24 h, both treatments produced only modest reductions in viability across the tested range; the IC_50_ was not reached for either formulation (NPs; 58.93% and free drug: 51.86% at 400 µg/mL). After 48 h, a clear concentration-dependent response was evident for both formulations, with the NPs producing stronger loss of viability at mid-high doses. Linear interpolation across the 50% threshold gave an estimated IC_50_ = 78.8 µg/mL for A549 NPs (48 h) and IC_50_ = 197.4 µg/mL for free QTN, indicating substantially higher potency for nanoformulations at the longer exposure time.

In H460 cells, the NPs also showed high potency, particularly after 48 h. At 24 h, the IC_50_ was not reached for the NPs whereas free QTN crossed 50% between 200 and 400 µg/mL, yielding IC_50_ = 322.3 µg/mL. After 48 h, both the treatments crossed the 50% threshold at lower concentrations, but NPs remained more potent. Estimated IC_50_ values were 56.5 µg/mL for H460 with NPs (48 h) and 69.7 µg/mL for H460 with free QTN. This improvement is consistent with well-recognized advantages of nanoparticulate delivery for poorly soluble polyphenols, including improved dispersion, increased cellular association/internalization and sustained intracellular exposure, which collectively translate into enhanced antiproliferative activity [42,43].

### 3.9. Proliferation Assay

**Figure 6:**
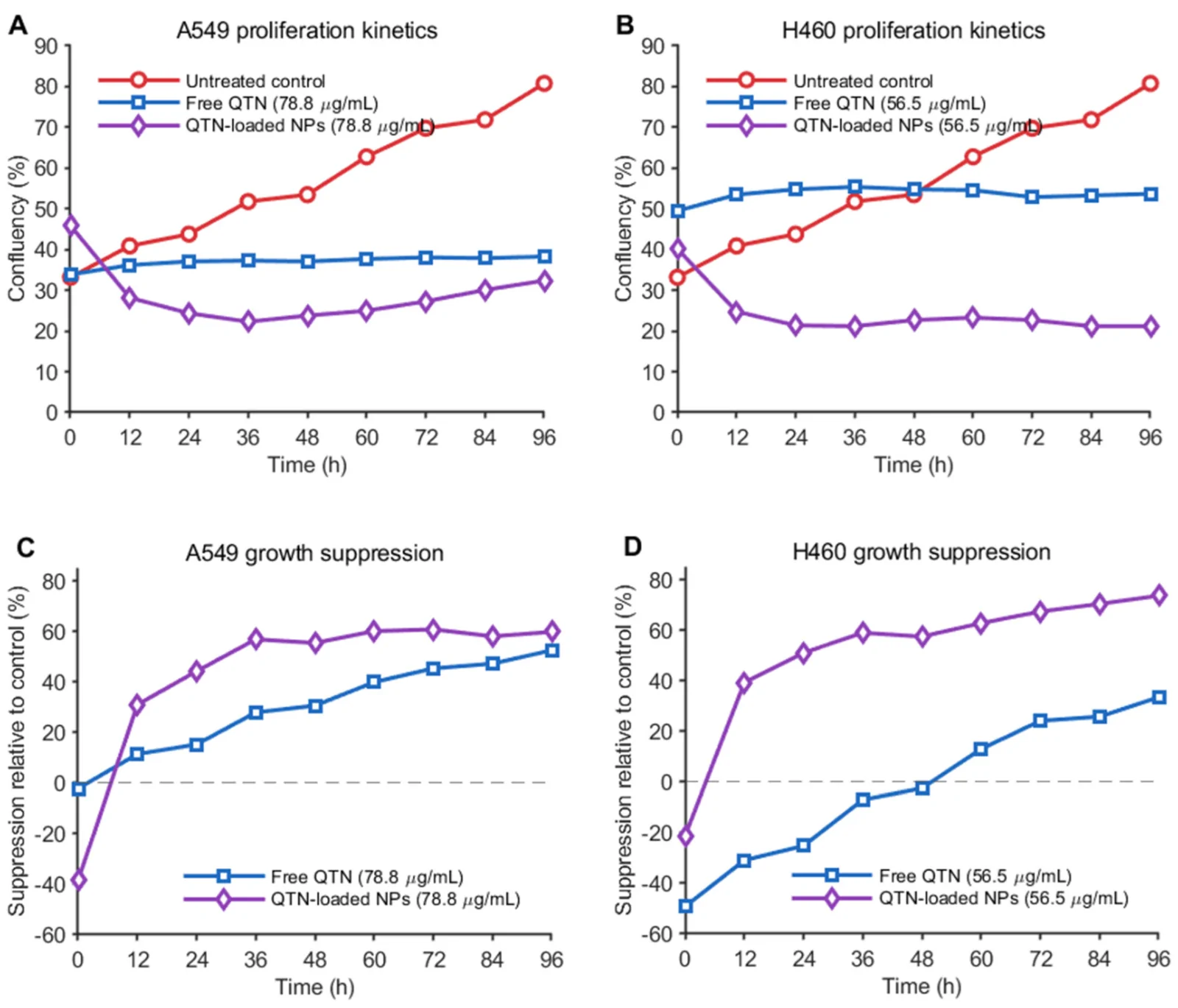
Time course of A549 and H460 cancer cell proliferation monitored for 4 days: (**A**) A549 proliferation kinetics; (**B**) H460 proliferation kinetics; (**C**) growth suppression in A549 cells; and (**D**) growth suppression in H460 cells.

Real-time confluence monitoring over 0–96 h was used to compare QTN-loaded nanoparticles with free QTN and untreated controls in A549 and H460 cells (Figure 6). At the tested dose, confluence remained lowered in the treated group than in untreated group over time, and the separation from untreated controls was consistently larger for the QTN-loaded formulation than for the free QTN. The difference between free QTN and loaded nanoparticles widened at later time points, where QTN-loaded nanoparticles produced the strongest suppression of confluence in both cell lines. These growth-inhibition patterns align with the viability data, indicating that the observed antiproliferative effect is attributable to QTN delivery rather than the nanoparticle matrix alone [6,22].

### 3.10. Cell Migration

**Figure 7:**
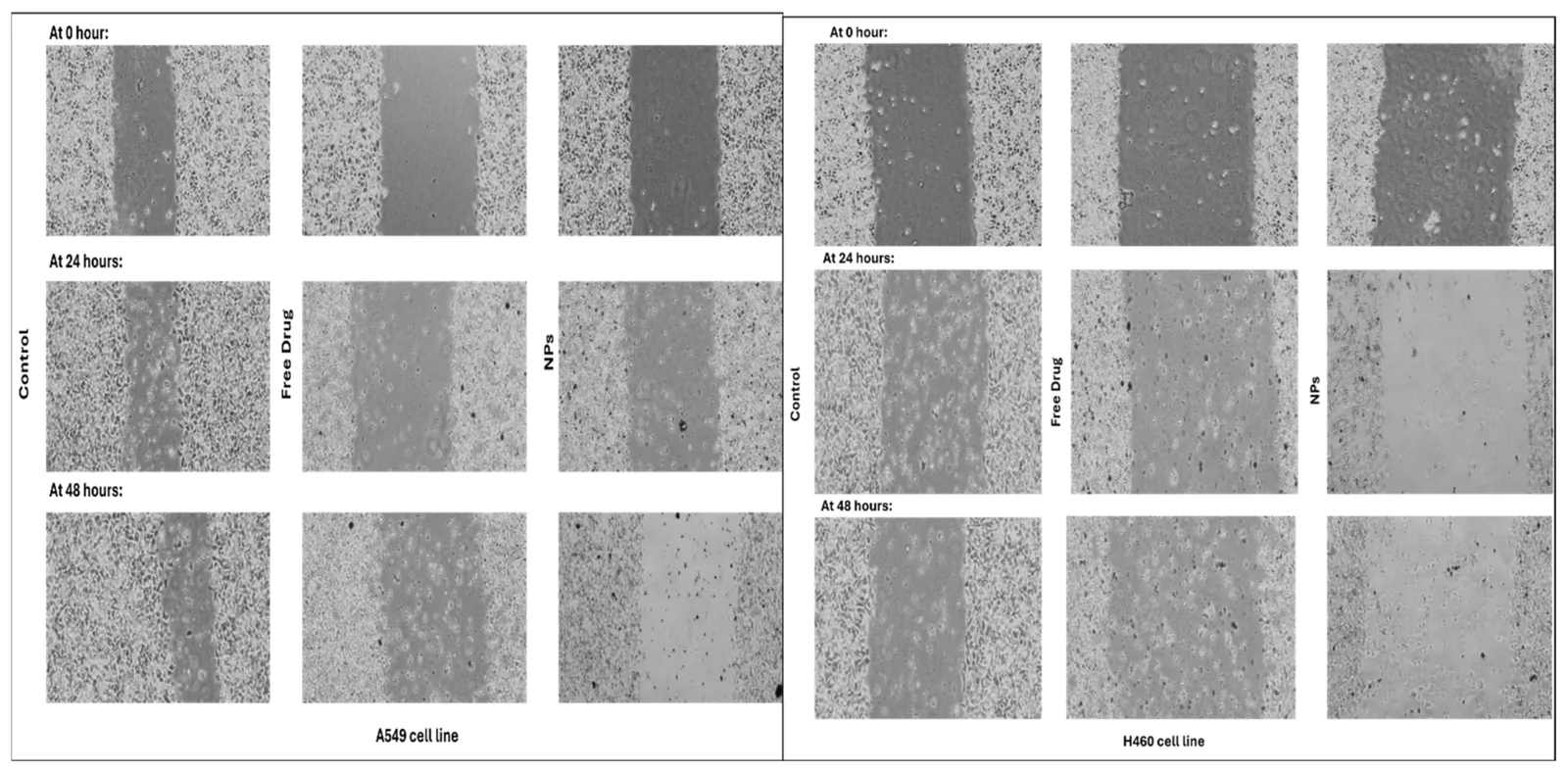
Effect of free QTN and QTN-loaded NPs on lung cancer cell (A549 and H460 cells) migration. (scale= 400 μm).

Cell mobility contributes to invasive behavior in lung cancer and suppression of migration is often used as an early functional indicator of anti-invasive potential. A scratch assay was used to evaluate whether QTN-loaded NPs modulate wound closure compared with free QTN in A549 and H460 monolayers. The confluent monolayers were scratched to generate a cell-free gap and then treated with IC_50_ concentrations of QTN in free and encapsulated forms alongside an untreated control. Images were acquired at 0, 24 and 48 h (Figure 7). In both cell lines, the control showed the expected time-dependent reduction in wound width, consistent with active lateral migration.

To the contrary, the treatment with QTN-loaded NPs produced a clear delay in gap repopulation in both cell lines, with the wound region remaining visibly more open at 24 h and persisting through 48 h relative to control. Free QTN also reduced wound closure compared with the control, however, the extent of closure inhibition appeared smaller than that observed with QTN-loaded NPs, as more cells advanced into the wound area by later timepoints. These observations suggest that the NP formulation enhances the apparent antimigratory activity of quercetin in A549 and H460 cells under tested conditions [44,45].

### 3.11. ROS Generation

**Figure 8:**
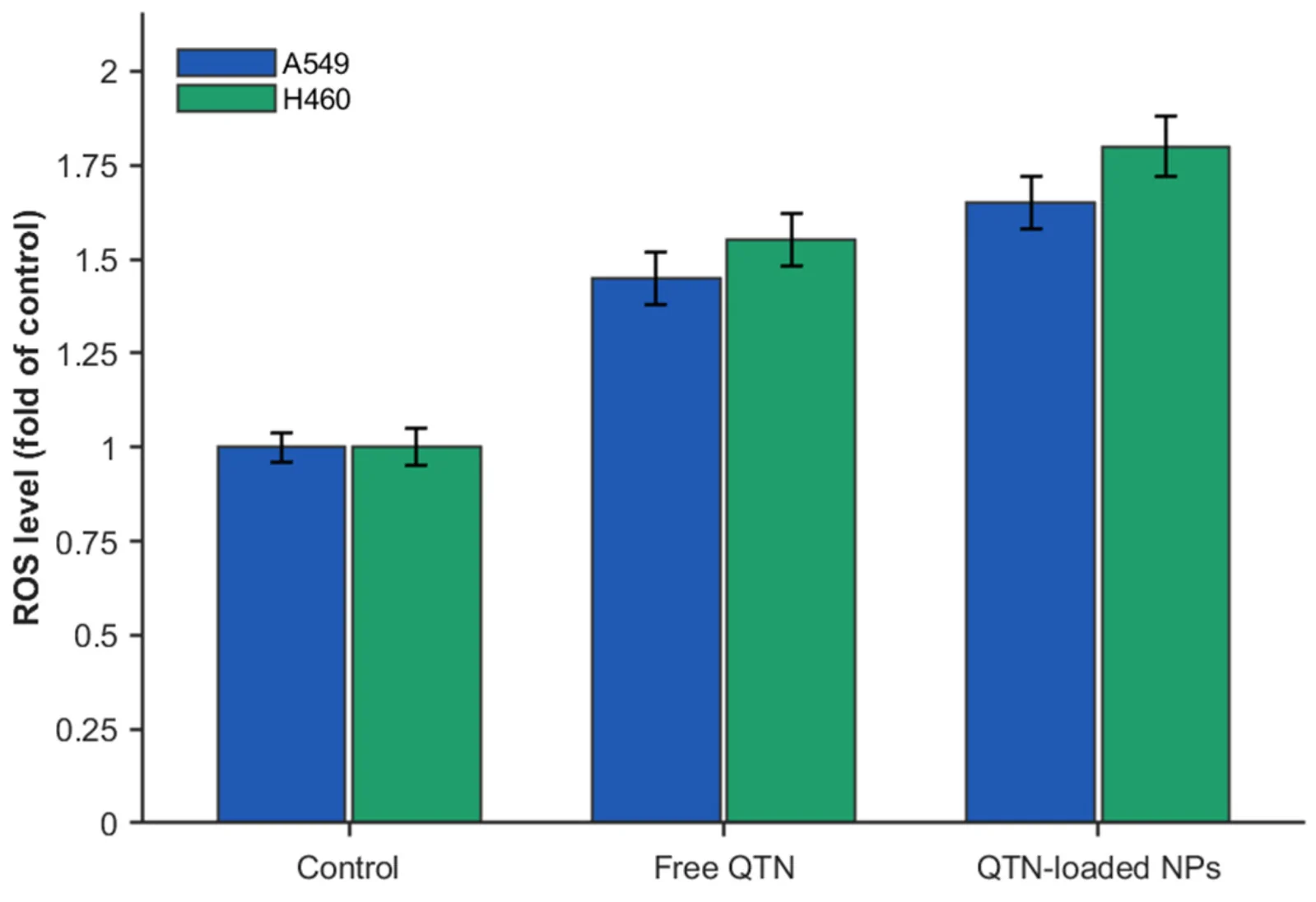
Lung cancer cell line (A549 and H460) response after 6 h’ incubation with free QTN versus QTN-loaded CtS/ShP NPs.

Intracellular reactive oxygen species (ROS) are a central regulator of redox homeostasis in cancer cells. When ROS level rises beyond the buffering capacity of antioxidant systems, oxidative stress can damage proteins, lipids and DNA and trigger apoptosis-related signaling pathways. Because many anticancer agents exert part of their efficacy through redox imbalance, the ROS production was quantified in A549 and H460 cells following exposure to QTN-loaded NPs. As shown in Figure 8, ROS levels were normalized to the untreated control (1.0-fold). Cells were treated with the respective IC_50_ value of QTN in free form or NP-loaded form. In both cell lines, free QTN increased ROS relative to control (A549 ~1.45-fold; H460 ~1.55-fold). Following QTN-loaded NP treatment, ROS reached ~1.65-fold in A549 and ~1.8-fold in H460, compared with the lower values for free QTN. These data support that CtS/ShP nanoencapsulation strengthens the ROS-associated fluorescence response to QTN under the tested IC_50_ exposure conditions [46,47].

### 3.12. Cellular Uptake of NPs

**Figure 9:**
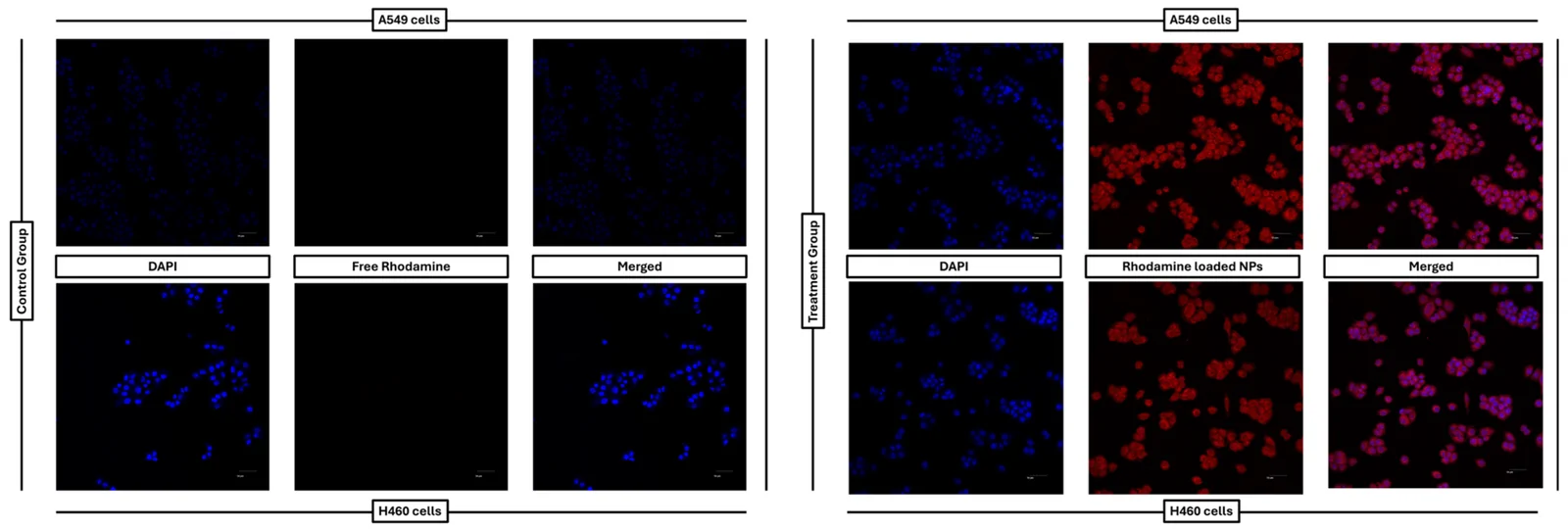
Fluorescence micrographs of the A549 and H460 cells after the cells were incubated with free rhodamine and rhodamine-loaded NPs for 4 h.

The fluorescent imaging performed after 4 h of incubation showed an efficient uptake of rhodamine-loaded CtS/ShP NPs by both A549 and H460 cell lines (Figure 9). In the untreated control group, only DAPI-stained nuclei were visible, whereas no detectable fluorescence was observed, confirming negligible background interference under the selected imaging conditions. In contrast, the cells that were treated with the rhodamine-loaded NPs showed a pronounced intracellular red fluorescence signal, thus indicating successful internalization of the NP formulation. In A549 cells, the rhodamine signal was largely cytoplasmic, with frequent perinuclear accumulation. The H460 cells showed the same distribution, with rhodamine’s red signal observed in most cells within the imaged fields. Relative to controls, NP-treated cells showed a marked increase in intracellular rhodamine signal in both A549 and H460. The localization trend is in line with internalization of the rhodamine-loaded CtS/ShP NPs under the tested conditions [39,48].

## 4. Conclusions

The development of CtS/ShP NPs was achieved as a natural polyelectrolyte delivery system, which displayed physicochemical and aerosol-performance characteristics relevant to pulmonary formulation fabrication. The nanoparticles were homogenized, positively charged, morphologically discrete, and showed high drug loading, acceptable powder-flow properties and biphasic release over 48 h. These characteristics imply that the chitosan–polysaccharide matrix offers colloidal stability as well as the controlled retention of quercetin. Nanoencapsulation of quercetin in vitro was linked to enhanced anticancer response in both A549 and H460 cells. The nanoparticle formulation, in comparison to free quercetin, was more effective in reducing cell viability, showed lower IC_50_ levels after 48 h, delayed wound healing, increased ROS-associated fluorescence, and displayed intracellular fluorescence consistent with definitive cellular uptake. A minimal cytotoxicity was displayed by the blank NPs under the tested conditions. Recent studies on anticancer flavonols have also linked their activity to oxidative stress modulation, apoptosis and suppression of metabolic signaling, while also showing that the nanoformulation can improve the delivery of poorly soluble flavonols [49]. In line with these findings, free QTN in the present study exerted antiproliferative, pro-oxidative and reduced scratch wound closure effects, whereas QTN-loaded CtS/ShP NPs produced a slightly stronger response. The minimal cytotoxicity of blank NPs further indicates that this enhancement was mainly associated with QTN rather than the non-specific carrier toxicity. Despite the in vitro assessment, the study presents proof-of-concept evidence for chitosan/*Salvia hispanica* polysaccharide nanoparticles as a natural-materials platform to deliver quercetin in lung cancer. Before translational potential can be defined, lung deposition, in vivo efficacy and safety must be determined.

## Figures and Tables

**Table 1:** Permutation importance and Garson’s sensitivity measurement.

Response	Variable	Permutation Importance (%)	Garson’s Importance (%)
Particle size (nm)	CtS (% *w*/*v*)	53.26	28.57
Particle size (nm)	ShP (% *w*/*v*)	19.77	27.3
Particle size (nm)	Formulation pH	20.88	22.02
Particle size (nm)	Stirring speed (rpm)	6.09	22.1
Encapsulation efficiency (%)	CtS (% *w*/*v*)	49.13	24.32
Encapsulation efficiency (%)	ShP (% *w*/*v*)	21.27	31.6
Encapsulation efficiency (%)	Formulation pH	19.89	18.36
Encapsulation efficiency (%)	Stirring speed (rpm)	9.76	25.7
Drug loading (%)	CtS (% *w*/*v*)	49.73	26.46
Drug loading (%)	ShP (% *w*/*v*)	21.01	30.75
Drug loading (%)	Formulation pH	20.40	20.01
Drug loading (%)	Stirring speed (rpm)	8.87	22.7

**Table 2:** ANN-model-predicted optimized formulation comparison with experimentally validated formulation.

Parameter	DdE-Predicted Formulation	ANN-Predicted Formulation	Experimentally Validated Formulation
Particle size (nm)	349.67	352.6	331 ± 14.3
Encapsulation efficiency (%)	76.87	76.5	73.8 ± 0.73
Drug loading (%)	15.6	15.5	14.20 ± 0.22
Desirability	0.99	0.97	-

## Data Availability

The original contributions presented in this study are included in the article/Supplementary Material. Further inquiries can be directed to the corresponding authors.

## Supplementary Materials

The following supporting information can be downloaded at: https://www.mdpi.com/article/10.3390/pharmaceutics18091095/s1, Table S1: Data set used for CDD and ANN modelling; Table S2: Screening of ANN architecture; Table S3: ANN performance metrics; Table S4: ANN descriptive statistical analysis; Table S5: Differential evolution fitness.
