# Supplementary material for: Aerosolized Quercetin-Loaded Chia Seed Polysaccharide Nanoparticles: Design of Experiments and Machine-Learning-Guided Optimization for Enhanced Lung Cancer Cell Delivery
Source: Pharmaceutics. 2026 Aug 30;18(9):1095. doi: 10.3390/pharmaceutics18091095 (PMC13610873; doi:10.3390/pharmaceutics18091095)
Supplement: Supplementary file 1 [file pharmaceutics-18-01095-s001.zip › pharmaceutics-4486540-supplementary.pdf]

Table S1: Data set used for CDD and ANN modelling.

No	Factor 1	Factor 2	Factor 3	Factor 4	Response 1		Response 2		Response 3	
1	X_1: Chitosan	X_2: Chia seed polysaccharide	X_3: pH	X_4: Stirring speed	Y_1: Particle size		Y_2: Encapsulation efficiency		Y_3: Drug loading	
2	% w/v	% w/v		rpm	nm		%		%	
					Actual	Predicted	Actual	Predicted	Actual	Predicted
3	0.02	0.05	5.8	600	389.8	388.61	67.81	67.22	10	10.05
4	0.035	0.035	5.15	1000	348.2	353.47	75.65	75.99	15.16	15.14
5	0.065	0.035	5.15	1000	365.9	367.99	72.47	72.78	13.02	13.16
6	0.035	0.035	6.45	1000	388.8	387.96	68.56	68.59	11.14	10.90
7	0.05	0.05	4.5	600	366.3	365.67	73.06	72.99	13.34	13.27
8	0.02	0.05	5.8	1400	396.2	398.07	65.87	65.84	9.04	8.99
9	0.02	0.02	4.5	1400	379.8	379.29	69.26	69.32	11.13	10.89
10	0.02	0.05	4.5	600	379.2	378.25	69.83	69.74	11.25	11.42
11	0.035	0.065	5.15	1000	379.4	378.95	69.17	68.88	10.68	11.02
12	0.035	0.035	5.15	1000	353.2	353.47	75.76	75.99	15.19	15.14
13	0.065	0.035	5.15	1000	368.9	367.99	73.06	72.78	13.5	13.16
14	0.05	0.02	5.8	1400	372.9	372.11	71.94	72.00	12.3	12.74
15	0.02	0.05	5.8	1400	397.1	398.07	65.64	65.84	8.97	8.99
16	0.035	0.005	5.15	1000	368.6	368.86	71.55	71.15	12.39	12.35
17	0.035	0.035	5.15	1000	355.3	353.47	75.85	75.99	15.03	15.14
18	0.005	0.035	5.15	1000	399.9	401.92	64.31	64.32	7.77	7.95
19	0.035	0.035	5.15	1000	357.6	353.47	76.15	75.99	14.94	15.14
20	0.05	0.05	5.8	600	372	372.62	71.29	71.29	12.17	12.26
21	0.035	0.035	3.85	1000	368.4	366.85	73.22	72.82	13.36	13.42
22	0.035	0.035	5.15	1000	355	353.47	75.67	75.99	15.46	15.14
23	0.035	0.035	5.15	1800	375.6	374.22	71.43	70.93	12.25	11.96
24	0.005	0.035	5.15	1000	404	401.92	64.54	64.32	7.86	7.95
25	0.035	0.065	5.15	1000	377.2	378.95	68.55	68.88	11.3	11.02
26	0.05	0.02	4.5	1400	361.1	361.36	73.38	73.71	13.97	13.89
27	0.02	0.02	4.5	600	378.6	375.24	69.9	70.17	11.7	11.73
28	0.035	0.035	5.15	1800	374.7	374.22	70.64	70.93	11.63	11.96
29	0.035	0.035	5.15	200	366	363.59	73.22	72.92	13.26	13.35
30	0.035	0.035	6.45	1000	387.2	387.96	68.67	68.59	10.8	10.90
31	0.05	0.05	5.8	1400	380.1	379.19	70.3	70.15	11.61	11.72
32	0.05	0.02	5.8	600	366.2	365.03	72.93	72.80	13.36	13.40
33	0.035	0.035	5.15	1000	351.6	353.47	76.13	75.99	15.23	15.14
34	0.05	0.02	4.5	1400	360.5	361.36	73.9	73.71	13.61	13.89
35	0.02	0.02	5.8	1400	390.5	393.45	66.62	66.79	9.27	9.37
36	0.035	0.035	5.15	1000	352.8	353.47	76.64	75.99	15.06	15.14
37	0.02	0.02	5.8	600	386.5	383.49	67.91	67.83	10.6	10.55
38	0.05	0.02	5.8	600	362.2	365.03	72.75	72.80	13.56	13.40

39	0.05	0.02	4.5	600	365	360.19	74.5	74.31	14.02	14.21
40	0.02	0.02	4.5	600	371.9	375.24	70.47	70.17	11.71	11.73
41	0.05	0.02	5.8	1400	371.9	372.11	71.96	72.00	13.04	12.74
42	0.02	0.02	5.8	1400	396	393.45	66.97	66.79	9.41	9.37
43	0.05	0.05	4.5	1400	369.1	366.33	71.77	72.05	13.26	13.06
44	0.05	0.02	4.5	600	358.6	360.19	74.09	74.31	14.44	14.21
45	0.02	0.05	4.5	600	377.1	378.25	69.55	69.74	11.79	11.42
46	0.02	0.05	5.8	600	389.3	388.61	66.8	67.22	10.29	10.05
47	0.02	0.05	4.5	1400	379.8	381.80	68.37	68.56	10.71	10.70
48	0.035	0.005	5.15	1000	369.3	368.86	70.98	71.15	12.3	12.35
49	0.02	0.02	4.5	1400	377.7	379.29	69.17	69.32	11.08	10.89
50	0.02	0.05	4.5	1400	386.2	381.80	68.7	68.56	10.58	10.70
51	0.05	0.05	5.8	600	374.7	372.62	71.29	71.29	11.99	12.26
52	0.05	0.05	4.5	1400	362.7	366.33	72.39	72.05	12.99	13.06
53	0.035	0.035	5.15	200	358.2	363.59	72.58	72.92	13.42	13.35
54	0.035	0.035	5.15	1000	354.1	353.47	76.1	75.99	15.04	15.14
55	0.05	0.05	4.5	600	366.2	365.67	72.94	72.99	13.25	13.27
56	0.05	0.05	5.8	1400	379	379.19	69.99	70.15	11.87	11.72

Table S2. Screening of ANN architecture.

Response	Hidden neurons	Architecture	Mean CV—R^2	SD CV—R^2	Median CV—R^2	Minimum CV—R^2	Maximum CV—R^2
Particle size (nm)	2	4/02/2001	0.415432	0.626441	0.676342	-1.56621	0.923337
Particle size (nm)	3	4/03/2001	0.27668	0.950984	0.671415	-3.07813	0.929437
Particle size (nm)	4	4/04/2001	0.453236	0.654389	0.68279	-1.73515	0.96061
Particle size (nm)	5	4/05/2001	0.031771	1.067513	0.604211	-2.95253	0.960313
Particle size (nm)	6	4/06/2001	0.26466	0.831182	0.568317	-2.32729	0.96063
Particle size (nm)	7	4/07/2001	0.298465	0.7083	0.692118	-1.32166	0.961466
Particle size (nm)	8	4/08/2001	0.649136	0.456998	0.802477	-1.23175	0.967627
Particle size (nm)	9	4/09/2001	0.19856	0.694144	0.311874	-1.47415	0.959984
Particle size (nm)	10	4/10/2001	0.309776	0.687229	0.424935	-1.90142	0.968903
Encapsulation efficiency (%)	2	4/02/2001	0.410026	0.464779	0.567105	-1.21689	0.78748
Encapsulation efficiency (%)	3	4/03/2001	0.161509	0.984817	0.470008	-3.40374	0.983647
Encapsulation efficiency (%)	4	4/04/2001	0.459928	0.587216	0.665481	-1.63013	0.986663
Encapsulation efficiency (%)	5	4/05/2001	0.155505	0.832306	0.493752	-1.95011	0.983507
Encapsulation efficiency (%)	6	4/06/2001	0.510696	0.492076	0.675752	-0.69417	0.984535
Encapsulation efficiency (%)	7	4/07/2001	0.417748	0.559936	0.535866	-1.5735	0.984855
Encapsulation efficiency (%)	8	4/08/2001	0.558099	0.460906	0.663579	-1.02586	0.985488
Encapsulation efficiency (%)	9	4/09/2001	0.690755	0.29183	0.756709	-0.04215	0.987404
Encapsulation efficiency (%)	10	4/10/2001	0.472626	0.460662	0.678129	-0.75855	0.985125
Drug loading (%)	2	4/02/2001	0.293704	0.492569	0.440015	-1.14735	0.789948
Drug loading (%)	3	4/03/2001	0.154319	0.934511	0.418605	-2.67836	0.973473

Drug loading (%)	4	4/04/2001	0.238853	0.879195	0.60319	-2.09143	0.99113
Drug loading (%)	5	4/05/2001	0.256453	0.707115	0.470017	-1.43516	0.990342
Drug loading (%)	6	4/06/2001	0.443792	0.590502	0.649415	-1.30627	0.990616
Drug loading (%)	7	4/07/2001	0.364431	0.559801	0.464908	-1.22145	0.990209
Drug loading (%)	8	4/08/2001	0.575668	0.46313	0.691904	-0.9056	0.990396
Drug loading (%)	9	4/09/2001	0.745901	0.281741	0.839216	-0.01203	0.989969
Drug loading (%)	10	4/10/2001	0.422882	0.57637	0.664437	-1.17646	0.989958

Table S3. ANN performance metrics.

Response	Architecture	Subset	N	R2	RMSE	MAE
Particle size (nm)	4/08/2001	Training	45	0.983947	1.771468	1.248395
Particle size (nm)	4/08/2001	Held-out test	11	0.813035	4.235397	3.347013
Encapsulation efficiency (%)	4/09/2001	Training	45	0.994878	0.236356	0.1798
Encapsulation efficiency (%)	4/09/2001	Held-out test	11	0.967354	0.414175	0.352941
Drug loading (%)	4/09/2001	Training	45	0.992479	0.175341	0.131615
Drug loading (%)	4/09/2001	Held-out test	11	0.984751	0.163193	0.148928

Table S4. ANN descriptive statistical analysis.

Variable	N	Mean	SD	Minimum	Q1 (25%)	Median (50%)	Q3 (75%)	Maximum	CV %
Chitosan (% w/v)	56	0.035	0.014013	0.005	0.02	0.035	0.05	0.065	40.03709
Chia seed polysaccharide (% w/v)	56	0.035	0.014013	0.005	0.02	0.035	0.05	0.065	40.03709
pH	56	5.15	0.607229	3.85	4.5	5.15	5.8	6.45	11.79086
Stirring speed (rpm)	56	1000	373.6795	200	600	1000	1400	1800	37.36795
Particle size (nm)	56	373.0339286	13.45048	348.2	363.75	371.95	379.875	404	3.605698
Encapsulation efficiency (%)	56	71.11196429	3.159357	64.31	68.6925	71.36	73.1	76.64	4.442792
Drug loading (%)	56	12.24625	1.923162	7.77	11.01	12.275	13.44	15.46	15.70409

Table S5. Differential evolution fitness.

Generation	Best fitness	Mean fitness
1	0.638354	0.570218
2	0.640423	0.600268
3	0.640423	0.613844
4	0.640423	0.62204
5	0.642711	0.630453
6	0.642711	0.632507
7	0.642723	0.63486
8	0.642723	0.636007
9	0.642737	0.638285

10	0.642952	0.640539
11	0.643014	0.640885
12	0.643014	0.641158
13	0.643026	0.641846
14	0.643026	0.64207
15	0.64313	0.642465
16	0.64313	0.642586
17	0.643136	0.642731
18	0.643136	0.642746
19	0.643136	0.642807
20	0.643136	0.642834
21	0.643136	0.642867
22	0.643136	0.6429
23	0.643136	0.642908
24	0.643136	0.642931
25	0.643136	0.642938
26	0.643136	0.642981
27	0.643136	0.642999
28	0.643136	0.643037
29	0.643136	0.643046
30	0.643138	0.64309
31	0.643138	0.6431
32	0.643139	0.64312
33	0.64314	0.643129
34	0.64314	0.643131
35	0.64314	0.643133
36	0.64314	0.643135
37	0.64314	0.643137
38	0.643141	0.643137
39	0.643141	0.643137
40	0.643141	0.643139
41	0.643141	0.64314
42	0.643141	0.64314
43	0.643141	0.64314
44	0.643141	0.64314
45	0.643141	0.64314

46	0.643141	0.643141
47	0.643141	0.643141
48	0.643141	0.643141
49	0.643141	0.643141
50	0.643141	0.643141
51	0.643141	0.643141
52	0.643141	0.643141
53	0.643141	0.643141
54	0.643141	0.643141
55	0.643141	0.643141
56	0.643141	0.643141
57	0.643141	0.643141
58	0.643141	0.643141
59	0.643141	0.643141
60	0.643141	0.643141
61	0.643141	0.643141
62	0.643141	0.643141
63	0.643141	0.643141
64	0.643141	0.643141
65	0.643141	0.643141
66	0.643141	0.643141
67	0.643141	0.643141
